# Mycophenolate Mofetil Enhances the Negative Effects of Sirolimus and Tacrolimus on Rat Kidney Cell Metabolism

**DOI:** 10.1371/journal.pone.0086202

**Published:** 2014-01-30

**Authors:** Jelena Klawitter, Jost Klawitter, Volker Schmitz, Touraj Shokati, Ekaterina Epshtein, Joshua M. Thurman, Uwe Christians

**Affiliations:** 1 Department of Anesthesiology, University of Colorado, Aurora, Colorado, United States of America; 2 Division of Renal Diseases and Hypertension, University of Colorado, Aurora, Colorado, United States of America; 3 Department of General-, Visceral- and Transplantation Surgery, Charité, Campus Virchow, Berlin, Germany; University of Florida, United States of America

## Abstract

**Background and Purpose:**

Mycophenolate mofetil (MMF) *per se* is not known to have negative effects on the kidney. MMF alone or in combination with sirolimus, can be the basis of calcineurin inhibitor (CNI)-free, kidney sparing drug protocols. However, long-term outcomes in patients on MMF/SRL seem to be inferior to those treated with regimens that include the CNI tacrolimus (TAC) due to an increased risk of allo-immune reactions. Interestingly, potential enhancement of the negative effects of SRL and TAC on the kidney by MMF has never been considered.

**Experimental Approach:**

It was our aim to study the effects of TAC, SRL and MMF alone and evaluate their interactions when combined on the rat kidney. For this purpose we used a comprehensive molecular marker approach including measurements of urinary 8-isoprostane concentrations (oxidative stress marker) and changes of urinary metabolite patterns (^1^H-NMR spectroscopy) and comparing these markers to renal function (glomerular filtration rate (GFR)) and morphologic alterations (histology).

**Key Results:**

While MMF alone did not impact GFR, its interaction with SRL and TAC led to a significant decrease of rats’ renal function. The decline went in parallel with a significant increase in urinary isoprostane concentrations and an enhancement of negative effects on urinary metabolite patterns.

**Conclusions:**

In broad summary, the present study showed that MMF may enhance the negative effects of TAC on kidney function and may even display nephrotoxic properties when combined with SRL.

## Introduction

The introduction of calcineurin inhibitors (CNIs) cyclosporine (CsA) and tacrolimus (TAC) in kidney transplantation has made transplantation a standard therapeutic intervention for end-stage renal diseases [1], [2]. Despite an improvement of short-term survival [3], long-term graft survival in renal transplant recipients has only seen a marginal improvement [4], [5]. Among others, the main complications arising from the CNI-treatment are their renal and vascular toxicity, diabetes, neurological disorders, infections and altered risk for malignancy.

When compared with CsA, TAC is associated with better blood pressure and lipid profile control [6]–[8], as well as better renal function [6], [7], [9], [10]. On the other hand, post transplantation diabetes may be considered the Achilles’ heel of TAC [11], [12].

In recent years, proliferation signal inhibitors such as sirolimus (SRL) and mycophenolate mofetil (MMF) have emerged as promising combination partners and alternatives to CNIs. Although SRL was originally believed to lack nephrotoxicity, it became evident in multicenter clinical trials that it may enhance CNI nephrotoxicity [13]. In contrast, MMF is commonly believed not to affect the kidney when used alone or in combination with CNIs. In addition, MMF does not seem to exhibit a negative impact on blood pressure, lipid profile, and/or glycemic metabolism [14], but it induces gastrointestinal toxicity. Thus the combination of low dose CNIs with MMF/MPA or SRL seems to be an attractive option for long-term maintenance immunosuppressive drug regimens after kidney transplantation [15]–[19].

Based on our pilot studies [20], [21], we used oral gavages of TAC, SRL and MMF doses that in rats resulted in blood concentrations within clinical ranges recommended for maintenance of kidney transplant patients TAC: 3–5 ng/mL, EVL: 3–8 ng/mL and MMF as mycophenolic acid (MPA): 2.5–4 µg/mL [22]–[26]. TAC and SRL are well-established substrates of active drug transporters such as P-glycoprotein and of cytochrome P450 drug metabolizing enzymes, most notably cytochrome P4503A, in the liver and small intestine [44]–[47]. Thus, it can be expected that competitive interactions at these drug transporters and drug metabolizing enzymes may cause pharmacokinetic interactions between MPA, TAC and SRL [27]–[30]. Our experimental setup included oral gavage of drugs over 28 days. The reason for this was to avoid triggering acute toxicity and cell death, and to characterize the effects of drug exposure on the metabolism of the kidney as reflected in urine excretion. We acknowledge that the salt-depleted rat model is the most often used model for the study of renal toxicity of immunosuppressants, however we deferred from it since salt-depletion alone already significantly affects kidney biochemistry [31].

Interestingly, to the best of our knowledge, it has never been considered that MMF/MPA may also enhance CNI and SRL nephrotoxicity. Therefore, it was our aim to study the effects of TAC, SRL and MMF alone and evaluate their interactions when combined on the rat kidney. For this purpose we used a comprehensive molecular marker approach including measurements of renal function (glomerular filtration rate (GFR)), kidney histology, urinary 8-isoprostane concentration (oxidative stress marker) and changes of urinary metabolite patterns (^1^H-NMR spectroscopy) [20], [21].

## Methods

### Animals

All animal protocols were approved by the University of Colorado Internal Animal Care and Use Committee in accordance with the National Institutes of Health guidelines (NIH publication No. 80–123). Ten- to fourteen-week-old male rats (Wistar Furth), weighing 280 to 330 g, obtained from Charles River Labs (Wilmington, MA), were housed in a temperature and light-controlled environment with access to tap water and food *ad libitum*. After at least two weeks of acclimatization, immunosuppressant treatment commenced.

### Drugs

Oral drinking solutions of sirolimus (Rapamune, Wyeth-Ayerst/Pfizer, Princeton, NJ), mycophenolate mofetil (CellCept, Roche, Nutley, NJ) and tacrolimus capsules (Prograf, Astellas Pharma, Deerfield, IL) were purchased from a local pharmacy. Drugs were administered daily by oral gavage in a constant volume according to group assignments. SRL and MMF were administered in the unmodified formulation (1 mg/mL); and contents of TAC capsules were suspended in skim milk (1 mg/mL) prior to administration. As aforementioned, all doses were chosen based on previous studies (23, 31, 32) and known to result in blood concentrations within or close to the range typically found in transplant patients. It should be noted that all drugs were administered orally and that the oral bioavailability of SRL and TAC in rats is markedly lower than in humans explaining the higher doses required to reach clinically relevant blood concentrations.

### Experimental Groups

Forty-five rats were randomly assigned to nine treatment groups (n = 5/group):

1. vehicle controls skim milk for 28 days
2. TAC1 1 mg/kg/day for 28 days
3. TAC3 3 mg/kg/day for 28 days
4. SRL1 1 mg/kg/d for 28 days
5. TAC1/SRL1 TAC 1 mg/kg/day+SRL 1 mg/kg/day for 28 days
6. TAC3/SRL1 TAC 3 mg/kg/day+SRL 1 mg/kg/day for 28 days
7. MMF20 20 mg/kg/day for 28 days
8. MMF20/TAC1 MMF 20 mg/kg/day+TAC 1 mg/kg/day for 28 days
9. MMF20/SRL1 MMF 20 mg/kg/day+SRL 1 mg/kg/day for 28 days

### Experimental Design

On day 27, rats were placed in metabolic cages for 24 h-urine collections. On the final day (day 28), two hours after receiving the final drug doses, animals were prepared for GFR measurements as described below. Animals were sacrificed to collect kidneys for histology and measurement of tissue drug concentrations, and whole blood for the determination of immunosuppressant drug concentrations. Plasma analysis for creatinine concentrations, blood urea nitrogen (BUN), alanine aminotransferase (ALT) and aspartate aminotransferase (AST) activities as well as measurement of creatinine concentrations in urine was performed by the University of Colorado Hospital (UCH) Clinical Laboratory using validated colorimetric (creatinine), conductivity-based (BUN) and coupled enzymatic (ALT, AST) methods. For more information about the tests, please refer to the test catalog of the UCH Clinical Laboratory: http://www.testmenu.com/public/cltdLaunch.aspx.

### Renal Function

Renal function was determined using the fluorescein isothiocyanate (FITC)-inuline method [32], [33]. Two hours after the final drug dosing, rats were placed on a thermostatically controlled surgical table and anesthetized by i.p. injection of ketamine (50 mg/kg)/xylazine (10 mg/kg) (KetaVed™/TranquiVed™, Vedco Inc., St. Joseph, MO). A 10-0 silicone catheter was inserted into the jugular vein for maintenance infusion. After injecting 2 mL of normal saline to provide sufficient intravascular volume, diluted (FITC)-inuline (Sigma, St. Louis, MO) (0.75 mg/100 mL saline) plus albumin (2.25 g/100 mL saline) were administered *via* perfusion pump for 2 hours at a rate of 2 mL/h as previously described by Lorenz and Gruenstein [33]. To monitor blood pressure throughout the experiment, a pressure transducer catheter (Millar Instruments, Houston, TX) was inserted into the carotid artery. After 1.5 hours of inulin infusion, a median laparotomy was performed, and a 10-0 silicone catheter was inserted into the left ureter. Urine was collected for 0.5 hours, and rats were sacrificed thereafter. Inulin concentration in plasma and urine was determined by fluorescence spectroscopy (Perseptive Biosystems Cytoflour Series 4000, Perkin Elmer, Walthma, MA). GFR values (µl/min) were calculated using the formula (UxV/P,) where U equals inulin concentration in urine, V is urine output over time and P is inulin concentration in plasma. For baseline correction, blank control plasma and urine samples were loaded with different concentrations of inulin and fluorescence absorption was recorded.

### Quantification of Immunosuppressants in Blood and Kidney Tissue

All drug concentrations were determined 4 hours after last dosing, when animals were sacrificed following renal clearance function measurements. For TAC and SRL measurements, whole blood samples were collected in heparinized tubes. For MPA, the active metabolite of MMF, heparin plasma was prepared following standard centrifugation procedures.

Flash-frozen renal tissue (100 to 200 mg) was mortared in liquid nitrogen and homogenized in 2 mL KH_2_PO_4_ buffer (pH 7.4). For protein precipitation, 800 µL methanol and 0.2 mmol/L ZnSO_4_ (80/20, v/v) were added to 200 µL of tissue homogenate or blood sample. Ascomycin (20 ng/mL; Sigma-Aldrich, St. Louis, MO) was added as internal standard for SRL and TAC [34], and MPA-d3 (Toronto Research Chemicals, Toronto, CA) served as internal standard for quantitation of MPA. All immunosuppressant drugs were quantified using a validated HPLC-MS assay [34], [35].

### Histology

For hematoxylin and eosin (H.E.) staining, kidney tissue samples were fixed in 10% buffered formaldehyde and embedded in paraffin, incubated for 5 minutes in Harris hematoxylin solution and for 60 seconds in eosin solution. Sections were washed with plain water, differentiated in 1% hydrochloric acid (HCl)+50% ethanol, and stain intensity was optimized in ammonia water. Finally, sections were rinsed in 70% ethyl alcohol and dehydrated in xylene solution.

### Semi Quantitative Scoring System

Images of the kidney sections were captured using a ScanScope Scanner Console and Aperio Image Scope software (APERIO Technologies, Vista, CA). Inflammatory infiltration of the kidneys was assessed by measurement of the density of nuclei using color saturation. In addition, 20 high-powered fields were examined in the cortical tubulointerstitium of each section. The number of fields containing tissue inflammation is reported as a percentage of the fields examined.

### ^1^H-NMR Spectroscopy

^1^H-NMR analysis of urine samples was performed using a Varian INOVA NMR 600 MHz spectrometer equipped with 5-mm HCN-PFG probe. Five hundred and fifty µL of urine were buffered with 73 µL 0.2 mol/L potassium phosphate buffer in D_2_O prior to analysis. The pH was finally adjusted to 5.65–5.75 with NaOD and DCl. To suppress water in urine, a standard Varian pre-saturation sequence was used. ^1^H-NMR spectra were obtained at 600 MHz using spectral width of 12 ppm and 32 K data arrays, and 64 scans with 90° flip angle. 14.8 sec was the D1 time, which was needed to fully relax all protons in the samples including the TMSP protons. Data analysis of the NMR data was performed using the MesTreC software version 4.4.1.0 (MesTreLab Research, Coruna, Spain). Drift correction, zero filling from 32 K to 64 K data points and a Gaussian window function were applied to the FID prior to Fourier transformation. Spectra were referenced to TMSP (0 ppm, trimethylsilyl propionic-2,2,3,3,-d4 acid dissolved in D_2_O to 50 mmol/L) as an external standard. Prior to integration, all ^1^H-NMR spectra were manually corrected for phase and baseline distortions. To compensate for differences in urine concentration, all spectra were normalized based on the total area of each urine spectrum [36], [37].

### Urinary 15-F_2t_-isoprostane Analysis

15-F_2t_-isoprostane is a stable marker of oxidative stress and it is well established that oxygen radicals play a key role in CNI toxicity alone and in combination with SRL (35). Urine samples were analyzed using a validated LC/LC-MS/MS method [38]. The resulting 15-F_2t_-isoprostane concentrations were finally normalized to the urinary creatinine concentrations.

### Statistical Analysis

All numerical data is presented as means±standard deviations. One-way analysis of variance (ANOVA) followed by the Holm-Sidak method as *post-hoc* test was used to determine group differences. The significance level was set at p<0.05 for all tests. Software used were SigmaPlot (version 11.0), SigmaStat (version 3.11, both by Systat Software, Point Richmond, CA, USA).

## Results

### Physiological Changes

While untreated and rats treated with 1 mg/kg/day of either SRL or TAC gained almost 30% weight in 28 days, combination treatments of SRL with TAC led to a significant weight loss with animals in the TAC3/SRL1 group losing more than half of their weight (Table 1). In regards to MMF treatment groups, animals seemed to only maintain their weight during the treatment period (Table 1).

**Table 1:** Percentage of weight gain and levels of liver function markers aspartate aminotransferase (AST) and alanine aminotransferase (ALT) in rats treated with immunosuppressants alone or in their combination for 28 days.

	Weight gain [%]	AST [U/L]	ALT [U/L]
Control	27.5	85.7±7.1	50.3±7.8
TAC1	21.4	117.8±16.8*	52.2±0.8
TAC3	−6.4	102.7±66.1	33.0±17.4
SRL1	29.4	76.3±8.2	34.5±5.7
TAC1/SRL1	−6.0	124.0±38.3*	40.5±11.9
TAC3/SRL1	−19.2	125.5±32.2*	42.5±5.3
MMF20	1.3	74.5±10.3	35.5±7.8
MMF20/TAC1	3.4	60.7±9.6**	27.5±3.3*
MMF20/SRL1	−1.3	69.0±10.8*	33.2±4.1*

All values are presented as means± standard deviations, n = 4–6/treatment group. Significance levels compared to either the untreated controls or among groups were determined using the one-way ANOVA in combination with a post-hoc pairwise multiple comparison (Holm-Sidak method): *p<0.05; **p<0.01.

### Serum Creatinine and BUN

**Figure 1:**
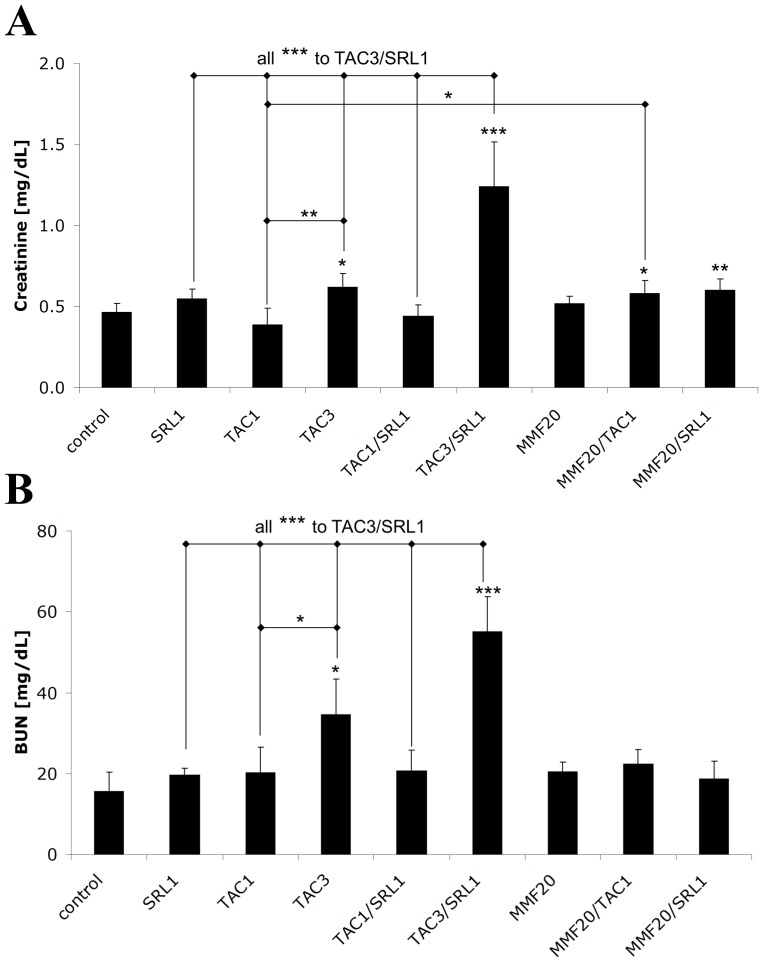
Changes in (A) serum creatinine and (B) blood urea nitrogen (BUN) concentrations of rats treated with different immunosuppressants alone or in their combination for 28 days. All values are presented as means± standard deviations, n = 5–10/treatment group. Significance levels compared to either the untreated controls or among groups were determined using the one-way ANOVA in combination with a post-hoc pairwise multiple comparison (Holm-Sidak method): *p<0.05; **p<0.01; ***p<0.001.

As previously observed [20], [21], treatment of rats with either 1 mg/kg/day SRL or TAC for 28 days did not cause significant changes in serum creatinine or BUN levels as compared to the controls (Figures 1A and 1B). However, after treatment with 3 mg TAC/kg/day for 28 days, BUN was more than 3-fold higher than in the controls (Figure 1B). Oral gavage of MMF alone did not change either serum creatinine or BUN (Figures 1A and 1B). But when MMF was combined with TAC or SRL, statistically significant increases of +25% and +29% of serum creatinine concentrations, respectively, were observed as compared to controls (Figure 1A).

In regards to urinary microalbumin excretion, no significant changes were observed.

While TAC1 and TAC+SRL treatments induced the serum activity of AST, combination of MMF with either TAC or SRL significantly decreased AST and ALT activities as compared to controls (Table 1).

### Glomerular Filtration Rates

**Figure 2:**
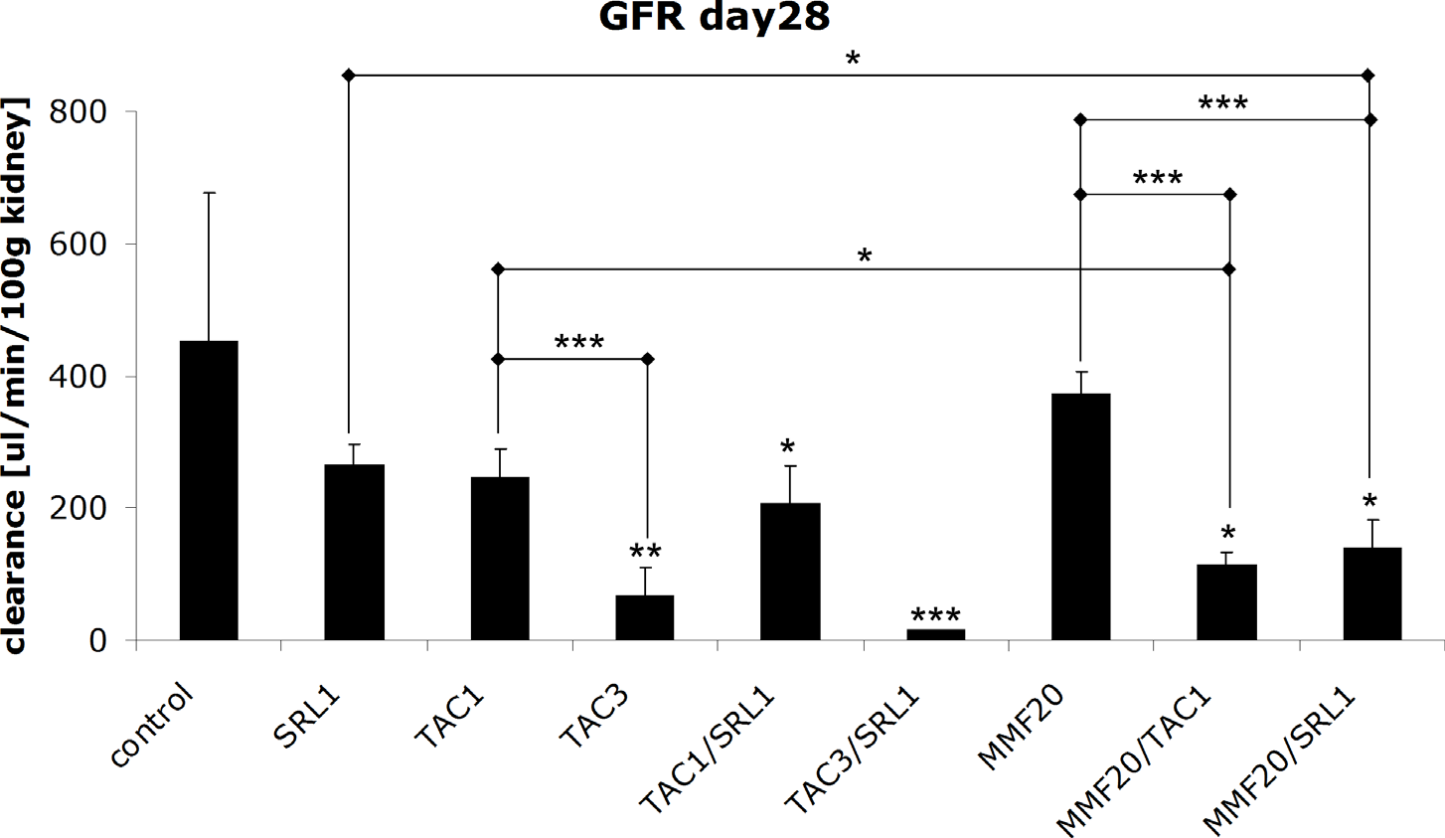
Changes in glomerular filtration rates (GFR) of rats treated with different immunosuppressant alone or in combination for 28 days. All values are presented as means± standard deviations, n = 4–9/treatment group). Significance levels compared to either the untreated controls or among groups were determined using the one-way ANOVA in combination with a post-hoc pairwise multiple comparison (Holm-Sidak method): *p<0.05; **p<0.01; ***p<0.001.

The inuline clearance rates in controls were not significantly different from those observed after 28 days in the TAC1 or SRL1 groups (Figure 2). Treatment with 3 mg/kg/day TAC as well as the combinations of TAC and SRL led to a significant decrease in GFRs (Figure 2). Interestingly, while MMF alone did not impact the inuline clearance of the kidney, its combination with TAC or SRL resulted in significant decreases (Figure 2).

### Histology

**Figure 3:**
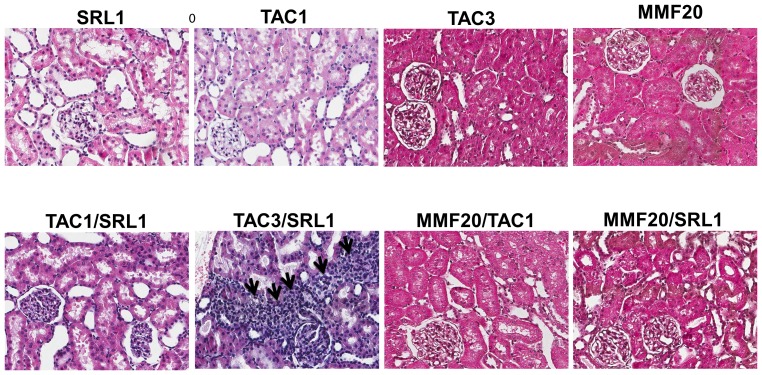
Histology of the different study groups (total number of tissue samples evaluated: n = 3/treatment group; all HE stain 50× magnified). Kidneys of TAC3/SRL1-treated rats showed large stripes of inflammation and tubular destruction, as well as monocytic tissue infiltration (presented with arrows). No significant changes in any of the other treatment groups were observed.

The kidneys of TAC3/SRL1-treated rats showed large stripes of inflammation and tubular destruction, which were visible on a low power view. Scaling into a high power view also demonstrated monocytic tissue infiltration (presented with arrows, Figure 3). Notably, no significant changes were observed in any of the other animal groups, except in one animal belonging to the TAC3 group.

### Blood and Tissue Drug Concentrations

**Figure 4:**
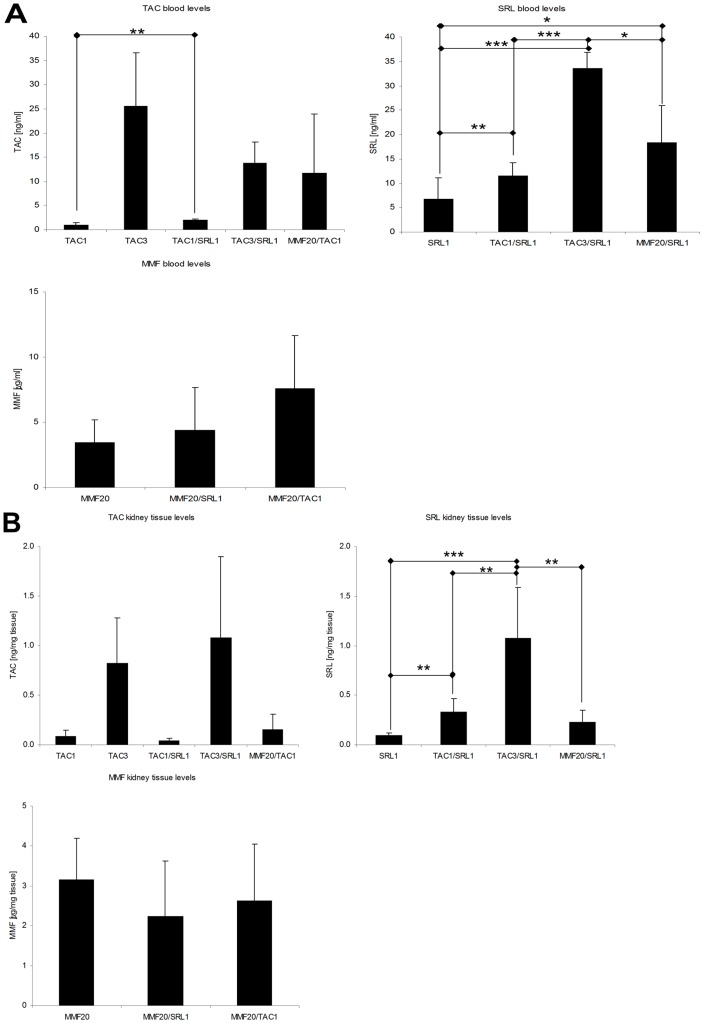
Blood (A) and (B) tissue concentrations of tacrolimus, sirolimus and mycophenolate mofetil (MMF; measured in the form of its hydrolyzed active metabolite mycophenolic acid (MPA)) four hours after the last dose as determined using a validated LC-MS/MS method. All concentrations are represented as means± standard deviations, n = 4–8/treatment group. Significance levels compared to either the untreated controls or among groups were determined using the one-way ANOVA in combination with a post-hoc pairwise multiple comparison (Holm-Sidak method): *p<0.05; **p<0.01; ***p<0.001.

When SRL and TAC where combined in TAC1/SRL1 group, TAC blood concentrations were higher than when given alone (Figure 4A). However, rats that received 3 mg/kg/day TAC did not show an augmentation in TAC concentrations by SRL, suggesting a threshold effect (Figure 4A). At the same time, SRL blood concentrations were higher when combined with TAC or MMF as compared to the single drug treatment (Figure 4A). Neither the combination with SRL nor TAC significantly changed MMF blood or tissue concentrations (Figures 4A and 4B).

In regards to TAC tissue concentrations after the 1 mg/kg/day dose, the concentrations seemed independent of the combination partner (Figure 4B). When TAC was dosed at 3 mg/kg/day, the SRL concentrations were more than 5-fold higher than in the kidneys of rats that received SRL (1 mg/kg/day) alone (Figure 4B). Other than in blood, MMF co-administration did not affect SRL concentrations in the kidney (Figure 4B).

### 15-F_2t_-isoprostane Concentrations in Urine

**Figure 5:**
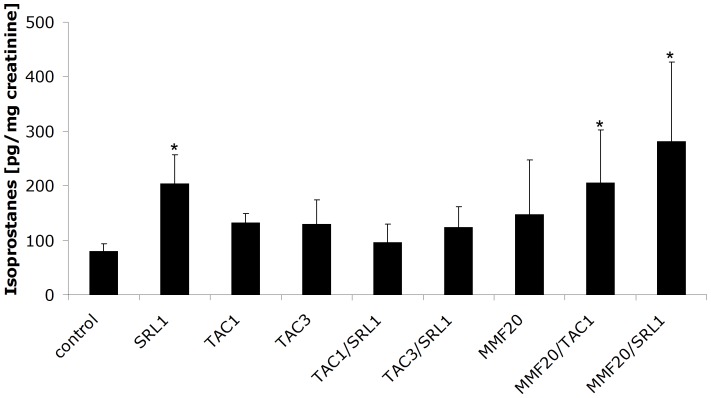
15-F_2t_-Isoprostane concentrations in urine of rats treated with different immunosuppressant alone or in combination for 28 days. All concentrations are presented as means± standard deviations, n = 4–8/treatment group. Significance levels compared to either the untreated controls or among groups were determined using the one-way ANOVA in combination with a post-hoc pairwise multiple comparison (Holm-Sidak method): *p<0.05.

Compared to untreated controls, isoprostane concentrations in urine were found to be higher in the following three treatment groups: SRL1, MMF20/SRL1 and MMF20/TAC1 (Figure 5).

### Metabolite Patterns in Urine as Assessed by ^1^H-NMR Spectroscopy

**Figure 6:**
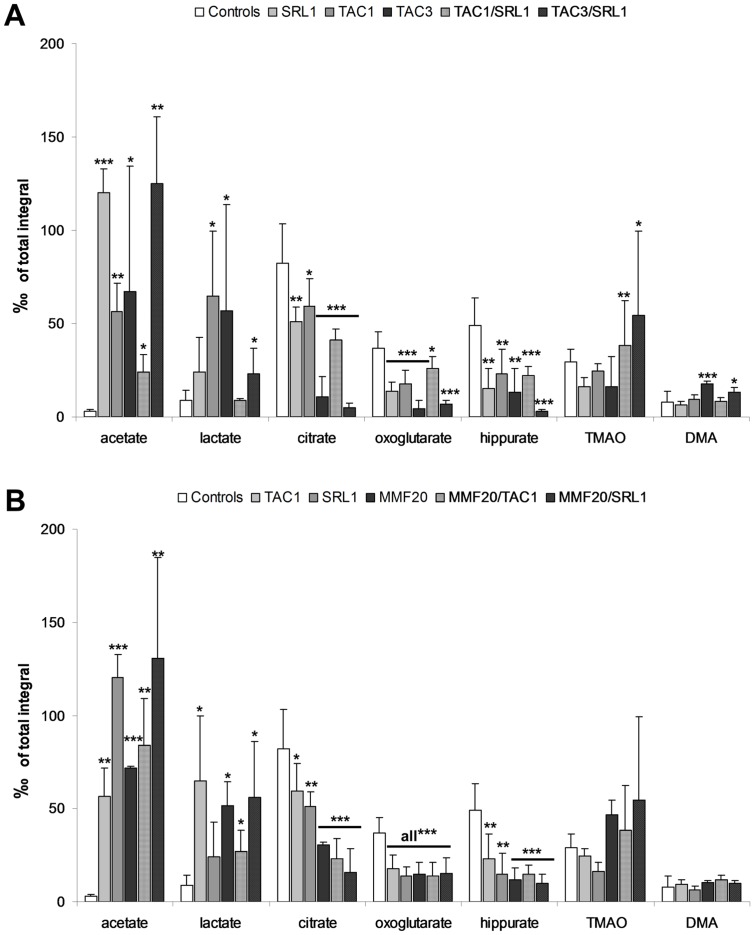
Changes in urine metabolite patterns after 28^1^H-NMR spectroscopy in (A) non-MMF containing regimens and (B) MMF-containing combination regiments. The pattern changes observed matched those typically associated with free radical formation [74] and S3 tubular damage [69]. All urine metabolites were normalized based on the total integral over the ^1^H-NMR spectra and are presented as means+ standard deviations with n = 4–5/treatment group. Significance levels to either the untreated controls or among groups were determined using the one-way ANOVA in combination with a post-hoc pairwise multiple comparison (Holm-Sidak method): *p<0.05; **p<0.01; ***p<0.001. Abbreviations: DMA: dimethylamine, TMAO: trimethylamine N-oxide.

Concentrations of urinary Krebs cycle intermediates citrate and 2-oxoglutarate were reduced in all treatment groups, most prominently in 3 mg TAC/kg/day (Figures 6A and 6B), in accordance with previously published results [20], [21], [26]. The excretion of the uremic toxin hippurate was also decreased as compared to controls (Figures 6A and 6B).

An increase in acetate concentration was observed under all therapies, whereas higher dimethylamine (DMA) and trimethylamine-oxide (TMAO) concentrations were seen in TAC3, TAC1/SRL1 and TAC3/SRL1 groups (Figure 6A). This increase is suggestive of impaired renal tubular function and medulla injury [39], [40].

Interestingly, while TAC alone increased urinary lactate excretion, addition of SRL antagonized the increase (Figure 6A). Urinary lactate was higher in all animals treated with MMF (Figure 6B).

## Discussion and Conclusions

With the known nephrotoxic properties of CNI, for the last 10 to 15 years, sirolimus and mycophenolate mofetil, two adjunctive non-CNI drugs, have been used in an attempt to lower concomitant maintenance CNI levels, possibly reducing graft attrition [15], [41]–[43]. Albeit less nephrotoxic, neither SRL nor MMF are side-effect free. While SRL provides acceptable protection from acute rejection [13], [44], its benefits are offset by concerns of synergistic nephrotoxicity with CNIs, hyperlipidemia, proteinuria, hematological toxicities, wound healing problems and increased risks of infection [13], [42], [43], [45]–[48]. Additionally, SRL may activate an innate immune response and shows toxic effects on glomerular endothelium and podocytes [49], [50]. MMF/MPA has been shown to reduce cell viability not only in CD4+ T cells but in human renal and cardiac cells as well [51]–[54]. Both drugs were shown to impair human β-islet cell function and survival [55]. In terms of combination treatments, a recent analysis evaluated outcomes of MMF in combination with SRL or TAC in solitary kidney transplant recipients transplanted between 2000 and 2005. This study showed that conditional graft survival in deceased donor kidney transplant recipients was significantly lower under SRL/MMF compared to patients on TAC/MMF immunosuppressive regimens at 5 years post-transplant [17]. Another study of 150 kidney transplant patients at 8-year follow-up confirmed that the maintenance therapy with TAC/MMF is more favorable than either TAC/SRL or CsA/SRL [56].

Current trends towards personalized immunosuppressive therapy require better strategies for avoidance of drug-related toxicity while maintaining efficacy [57]–[59]. In our previous studies, we had shown that one of the reasons for the enhancement of CsA toxicity by SRL may be a toxicokinetic interaction leading to increased CsA kidney tissue concentrations in the presence of SRL [20], [60]. In an attempt to better understand the effects of TAC and SRL interactions with MMF on changes in renal function and cell metabolism, we designed our study in normally fed rats and with drug doses leading to blood (TAC, SRL) and plasma concentrations (MPA) similar to the target clinical therapeutic ranges in patients [22]–[26].

After 3 mg/kg/day TAC alone and in combination with SRL, rats exhibited increased serum creatinine and BUN levels, decreased GFR and histomorphologic alterations in the kidney (tubular vacuolization, tubular epithelial damage), all changes considered typical for clinical CNI toxicity [61]. Interestingly, both, SRL and MMF, are clinically not considered to display any nephrotoxic properties, and as previously discussed, the slope of GFR decline per month is flatter in the TAC/MMF than in the TAC/SRL group [56], [62], [63]. Therefore, our results for single drugs were in accordance with clinical observations. However, MMF combination treatments (with TAC and SRL) significantly reduced the GFR, even below those in TAC1/SRL1 groups. Our rat model might not best represent the clinical situation. However, we observed toxicity synergisms between MMF and SRL/TAC on the kidney, and this deserves to be investigated in future mechanistic studies.

In regards to the toxicokinetic interactions, no significant changes in TAC kidney concentrations were observed in the present study. This basically excludes an interplay between intracellular TAC tissue concentration and renal function. In contrast, accumulation of SRL might potentiate the negative effects of TAC and MMF on the kidney, since SRL blood and tissue concentrations were higher when it was combined with either TAC or MMF.

Our previous studies suggested that the negative effects of CNIs and their enhancement by SRL are partially mediated by a decrease in mitochondrial energy metabolism, accompanied with an increase in reactive oxygen species (ROS) [20], [21], [34], [64], [65]. Although an increase in oxidative stress marker 15-F_2t_-isoprostane [66]–[68] is also observed in some of the study groups, the rise did not seem to parallel the reduction in GFR in all animals. Possibly, in animals treated with TAC/SRL combinations for 28 days, we might have been past the phase of detectable oxidative stress, and these changes might have been adapted for.

^1^H-NMR is an established tool to detect specific changes of urine metabolite patterns and these could be correlated with specific histopathological changes induced by a variety of nephrotoxins [69]–[71]. In our previous animal studies, CNI-induced proximal tubular injury was associated with reduction of urinary concentrations of Krebs cycle intermediates whereas urinary concentrations of trimethylamine-N-oxide (TMAO), acetate, lactate, trimethylamine and glucose increased [20], [21], [64]. Co-administration of SRL enhanced these negative effects. In the present study, urinary metabolite pattern changes were similar to those observed in previous studies (31,32,43), with increased acetate and lactate levels and reduced concentrations of Krebs cycle intermediates in TAC/SRL combination groups. And while treatment of rats with MMF alone did not lead to significant changes in terms of GFR and isoprostanes, it had a clear impact on urine metabolite patterns. Surprisingly, in comparison to treatment with TAC and SRL alone, co-administration of MMF was not only associated with a significant further increase in oxidative stress marker, but also with a further significant reduction of Krebs cycle intermediates and an increase of urinary lactate and acetate concentrations. Most importantly, co-administration of MMF (20 mg/kg) caused a significant reduction of GFR compared to SRL1 and TAC1.

In the present study, we used an established strategy that allowed for studying early effects of immunosuppressants and their combinations on kidney cell metabolism before or while only mild changes in kidney histology occurred. A key feature of this rat nephrotoxicity model was that immunosuppressant drugs were dosed so that ideally no histological changes occur during the observation period. Typically, histological damage goes parallel with secondary responses such as inflammation that *per se* already change cell and urine metabolite patterns. This means that they cause “metabolic noise” that makes it difficult, if not impossible, to discern which of the metabolic changes are specific for the study drugs. We were able to demonstrate that the monitoring of metabolites in urine such as isoprostanes and/or Krebs cycle intermediates may be more sensitive than creatinine concentrations in serum. A large body of literature is available that has shown an association between changes in urine metabolite patterns and drug toxicity as confirmed by histological changes. Changes in urine metabolite patterns as assessed by NMR spectroscopy have extensively been used for the evaluation of kidney region specific toxins [72], [73].

Our study surprisingly showed that MMF and SRL in combination have significant negative effects on the rat kidney and, in contradiction to present clinical opinion, MMF may also enhance the negative effects of TAC on the kidney reflected by a reduction of GFR, which is accompanied by induction of oxidative stress and by causing further changes in urinary metabolites indicative of tubular injury. Although it has to be taken into consideration that this study was conducted in healthy rats and that some of the results could not translate into changes typically associated with clinical nephrotoxicity, the present study nevertheless shows that MMF has the potential to enhance the negative effects of TAC and SRL on the kidney and that drug-drug interactions may be a contributing factor to the relatively poor long-term clinical outcome of patients on MMF/SRL immunosuppressive drug regimens. Hence, the results of the present study provide the rationale for further follow-up.
